# Array Comparative Genomic Hybridisation and Droplet Digital PCR Uncover Recurrent Copy Number Variation of the *TTN* Segmental Duplication Region

**DOI:** 10.3390/genes13050905

**Published:** 2022-05-19

**Authors:** Lydia Sagath, Vilma-Lotta Lehtokari, Katarina Pelin, Kirsi Kiiski

**Affiliations:** 1 Folkhälsan Research Center, Biomedicum, 00290 Helsinki, Finland; vilma.lehtokari@helsinki.fi (V.-L.L.); katarina.pelin@helsinki.fi (K.P.); 2 Department of Medical Genetics, Medicum, University of Helsinki, 00014 Helsinki, Finland; 3 Molecular and Integrative Biosciences Research Programme, Faculty of Biological and Environmental Sciences, University of Helsinki, 00014 Helsinki, Finland; 4 Laboratory of Genetics, Division of Genetics and Clinical Pharmacology, HUS Diagnostic Center, University of Helsinki and Helsinki University Hospital, 00029 Helsinki, Finland

**Keywords:** Droplet Digital PCR, titin, *TTN*, copy number variation, segmental duplication

## Abstract

Intragenic segmental duplication regions are potential hotspots for recurrent copy number variation and possible pathogenic aberrations. Two large sarcomeric genes, nebulin and titin, both contain such segmental duplication regions. Using our custom Comparative Genomic Hybridisation array, we have previously shown that a gain or loss of more than one copy of the repeated block of the nebulin triplicate region constitutes a recessive pathogenic mutation. Using targeted array-CGH, similar copy number variants can be detected in the segmental duplication region of titin. Due to the limitations of the array-CGH methodology and the repetitiveness of the region, the exact copy numbers of the blocks could not be determined. Therefore, we developed complementary custom Droplet Digital PCR assays for the titin segmental duplication region to confirm true variation. Our combined methods show that the titin segmental duplication region is subject to recurrent copy number variation. Gains and losses were detected in samples from healthy individuals as well as in samples from patients with different muscle disorders. The copy number variation observed in our cohort is likely benign, but pathogenic copy number variants in the segmental duplication region of titin cannot be excluded. Further investigations are needed, however, this region should no longer be neglected in genetic analyses.

## 1. Introduction

Segmental duplications (SD) are highly identical, 10–300 kb long genomic sequences present from two to a few times in the genome, either interspersed or in tandem [1,2,3]. They predispose regions to copy number variants (CNVs) and may thus act as mutational hotspots [4,5,6].

**Figure 1:**
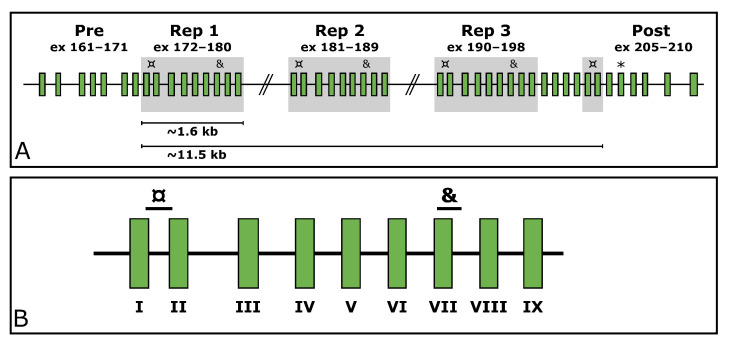
The structure of the *TTN* SD region. (**A**) shows the repeated blocks shaded in grey. The custom ddPCR assays targeting the *TTN* SD region exons I and VII are marked with ¤ and & respectively. The targeted exons downstream of the segmental duplication are marked with an asterisk (*). (**B**) shows a zoom-in on the repeated block along with the target locations of the assays. The exons of the repeated block have been assigned Roman numerals for simplification; e.g., exon I represents the first exon of each block (172/181/190/203), and exon VII represents the seventh exon of each block (178/187/196).

Titin (*TTN*, MIM ID *188840) is a gargantuan gene highly expressed in skeletal muscle. According to the reference sequence of the longest *TTN* transcript (ENST00000589042.5, CCDS59435.1), it consists of 363 exons. In its middle, it holds a region encoding domains rich in proline (P), glutamate (E), valine (V), and lysine (K), referred to as the PEVK region [7]. Within the PEVK region, it withholds a SD region (exons 172–180, 181–189, 190–198, and 203–204). This region consists of a 9-exon-block repeated three times, after which the two first exons of the block appear a fourth time. These two exons are separated by four exons from the last exon of the last repeated block (exon 198). The structure of the *TTN* SD region is depicted in Figure 1.

Mutations in *TTN* can cause several different neuromuscular diseases, such as tibial muscular dystrophy and cardiomyopathies, in both recessive and dominant inheritance modes (MIM IDs dilated cardiomyopathy #604145, familial hypertrophic cardiomyopathy #613765, limb-girdle muscular dystrophy type 2J #608807, proximal myopathy #603689, Salih myopathy #611705, and tibial muscular dystrophy #600334) [8,9,10,11,12].

The standard methods for routine CNV analysis are still microarray-based technologies, either using SNP or CNV probes, such as array Comparative Genomic Hybridisation (aCGH). However, CNV analysis methods based on massively parallel sequencing (MPS) data are rapidly improving in accuracy and reliability [13,14,15,16]. SDs and other repetitive regions still challenge both aCGH and methods based on MPS in CNV detection. Designing unique CNV or SNP probes is challenging due to the repetitive nature of these regions. Thus, these regions are typically avoided in commercial aCGH designs and often left with minimal to no probe coverage. Similarly, the alignment of short sequences challenges the analysis of repetitive regions with MPS-based methods.

We have previously designed and published two validated custom tiling CGH-arrays for the detection of CNVs in neuromuscular disorder genes [17,18]. These arrays also cover the *TTN* SD and a similar SD in another muscle gene, the triplicate (TRI) region in nebulin (encoded by the *NEB* gene, MIM ID *161650), to shed light on these regions and their variations [17,18].

Like *TTN*, *NEB* is a large structural protein highly expressed in skeletal muscle. Pathogenic variants in *NEB* are a known cause of muscle disorders, such as nemaline myopathy (MIM #256030). Both *TTN* and *NEB* are thought to act as molecular templates, or rulers, for muscle filament length and structure [19,20,21,22,23,24]. As per this Ruler Hypothesis, large enough gains and losses in CN in the *NEB* TRI and *TTN* SD regions may be pathogenic [19,20,21,22,23,24,25,26]. It has been shown that gains of two blocks of *NEB* TRI in one allele are, in fact, disease-causing [25].

From a methodological molecular diagnostic perspective, the major difference between the SD regions of *NEB* and *TTN* is the difference in size—the *NEB* TRI covers altogether 30 kb of genomic region, which is roughly three times more than the *TTN* SD. Despite its repetitiveness, its length allows a tiled aCGH design in this region.

To allow for large-scale screening of CNVs of the *NEB* TRI region, we previously developed two custom Droplet Digital PCR (ddPCR) assays targeting the region [27]. Here, we present the extension of the study; custom ddPCR assays for the detection of CNVs within the *TTN* SD region. The study aimed to use custom ddPCR assays to validate CNVs of the *TTN* SD region in a cohort consisting of samples from neuromuscular disorder patients and family members previously studied using a custom CGH-array [18]. Using ddPCR and aCGH data, we show that the *TTN* SD region is subject to CNVs in a similar fashion to the *NEB* TRI region. To our knowledge, this is the first publication acknowledging CNVs within *TTN* SD to this degree and shows that this region should no longer be neglected in genetic analyses.

## 2. Materials and Methods

### 2.1. Samples

Altogether, 62 samples from 42 neuromuscular disorder families were acquired for the study. Of these, 42 were index patient samples, and the remaining 20 samples were from unaffected family members. The patient phenotypes included nemaline myopathy (*n* = 18), distal nemaline myopathy (*n* = 2), asymmetric distal myopathy (*n* = 1), cap myopathy (*n* = 1), unspecified congenital myopathy with arthrogryposis (*n* = 1), and unspecified congenital myopathy (*n* = 12). Nine of the patients had previously received a final molecular genetic diagnosis. Causative CNVs of the *TTN* SD region were not expected in the cohort.

The DNA stocks had been extracted either from peripheral blood or from saliva, eluted into EDTA, TE-buffer, or water, and stored at −20 °C. The DNA concentration and quality were checked with DeNovix DS-11 FX+ Spectrophotometer/Fluorometer (DeNovix Inc., Wilmington, DE, USA). Subsequent dilutions for the ddPCR reactions were performed in sterile water and stored at 4 °C.

### 2.2. Comparative Genomic Hybridisation Array Design, Protocol, and Analysis

All samples were run using a custom CGH-array for neuromuscular disorders (NMD-CGH-array) as previously described [18].

The aCGH data were manually aligned for *TTN* and *NEB* to gain a zero baseline to avoid any subtle differences caused by the genome-wide normalisation of the analysis software (CytoSure Interpret Software v.4.11.30, Oxford Gene Technology Ltd, Cambridge, UK). The log_2_ value for the *TTN* SD region and large regions (124 kb and 157 kb) of the *TTN* gene upstream and downstream of the SD were extracted from the aCGH data. The breakpoints used for normalisation of *TTN* aCGH results were Chr2:(179389578_179390615)_(179512818_ 179513536) (upstream backbone), Chr2:(179533543_179533609)_(179691400_179690759) (downstream backbone), and Chr2:(179518163_179518846)_(179528302_179528492) (SD region). The breakpoints used for the normalisation of *NEB* have been published earlier [27].

The genomic locations for the aCGH data are given in the reference genome build Hg19/GRCh37. The normalised log_2_ value of the *TTN* SD region was acquired by subtracting the averaged background log_2_ value from the log_2_ value of the *TTN* SD region. The CNs of the SD region and the *TTN* backbone were estimated by converting the log_2_ values of the normalised *TTN* SD region and the average background log_2_ values to CNs assuming normal CNs of six and two, respectively.

### 2.3. Droplet Digital PCR

The ddPCR assays were designed, performed, and analysed according to the dMIQE guidelines [28,29]. The dMIQE checklist is available in Supplemental Table S1.

#### 2.3.1. Primer and Probe Design

Custom assays were designed for three regions in *TTN* (NG_011618.3). Two of these target repeated exons within the SD region, and one targets an exon located downstream from the SD region (exon 206, Figure 1A). The assays targeting the SD span from the end of exon 172/181/190/203 to the beginning of exon 173/182/191/204 (hereinafter referred to as *TTN* SD exon I as per the *TTN* SD exon it begins in) and from the beginning of exon 178/187/196 to the beginning of intron 178/187/196 (hereinafter referred to as *TTN* SD exon VII) (Figure 1B). The assay targeting the exon 206 downstream of the SD region is hereinafter referred to as *TTN* Post-SD.

Primers and probes for the assays were designed using Primer3Plus (v.3.2.4, https://www.primer3plus.com/index.html, accessed 17 May 2021) [30,31,32]. Primers were designed to have a melting temperature (Tm) of approximately 60 °C, a GC content of 50–60%, a length of 20 bp, and avoiding putative secondary structures and G or C repeats over three bases long. The amplicons were not allowed to contain the BsuRI cut site sequence (GGCC) or the EcoRV cut site sequence (GATATC). Amplicon lengths vary from 101 to 120 bp.

Hydrolysis probes were designed to have a Tm of approximately 65 °C and a GC content of 30%–80%, aiming for a length of 25 bp. Custom probes were labelled with fluorescein amitide (FAM). Tm was calculated by the nearest neighbour method using OligoCalc (v.3.27, http://biotools.nubic.northwestern.edu/OligoCalc.html, accessed 17 May 2021) [33].

An in silico specificity screen was performed using the Standard Nucleotide BLAST blastn suite (v.2.11.0, https://blast.ncbi.nlm.nih.gov/Blast.cgi, accessed 17 May 2021) [34,35], allowing four hits for the *TTN* SD exon I assay primers and probes, three hits for the *TTN* SD exon VII assay, and one hit for the *TTN* Post-SD assay. The hits were confirmed to represent the respective targeted regions within *TTN*.

All custom primer and probe sequences, along with amplicon lengths and locations within the reference sequences NG_011618.3 and NG_009382.2 are presented in Supplemental Table S2. The reference used was a commercial *EIF2C1* ddPCR probe assay labelled with hexachloro-fluorescein (HEX) (cat. no. 10031243, Bio-Rad Laboratories Inc., Hercules, CA, USA). *EIF2C1*, also known as Argonaute 1 (*AGO1*), is a diploid gene located in the chromosomal region 1p34.3.

The previously published custom ddPCR assay for *NEB* TRI exon VIII [27] was included as a positive control for each sample in every run.

All assays were obtained from Bio-Rad Laboratories, Inc. (Hercules, CA, USA) as custom ordered primer/probe assays at a primer:probe ratio of 3.6:1. The concentrations were 900 nM of primer and 250 nM of probe in the final reaction mix.

#### 2.3.2. Optimisation of ddPCR Assay Conditions

The optimal melting temperature for the assays was determined by running the reactions in a gradient PCR with different melting temperatures. The assays were evaluated using the melting temperatures of 57.5, 58.0, 58.5, 59.0, 59.5, 60.0, 60.5, and 61.0 °C. A melting temperature of 59.5 °C was chosen as it gave an adequate separation between the droplet clusters in all assays.

#### 2.3.3. Assay Protocol

The total reaction volume was 20 μL, consisting of 10 ng of genomic template DNA in a volume of 7 μL, 1 μL of custom and reference assay each, 1 μL of restriction enzyme mix, and 10 μL of 2x ddPCR Supermix for Probes (No dUTPs) (cat. no. 1863023, Bio-Rad Laboratories Inc.). The restriction enzyme mix contained equal amounts of BsuRI (cat. no. FD0154, Thermo Scientific, Waltham, MA, USA) and EcoRV (cat. no. F0304, Thermo Scientific) for the *TTN* assays, and 1:1 diluted BsuRI in 10x FastDigest Buffer (cat. no. B64, Thermo Scientific) for the *NEB* assay. For each reaction, 22 μL of reaction mix was prepared, of which 20 μL was pipetted onto the DG8 Cartridge (cat. no. 1864008, Bio-Rad Laboratories Inc.). The cartridges were covered with DG8 Gaskets (cat. no. 1863009, Bio-Rad Laboratories Inc.).

The reactions were divided into approximately 1 nl droplets using the Droplet Generator QX2000 (Bio-Rad Laboratories Inc.) with Droplet Generator Oil (cat. no. 1863005, Bio-Rad Laboratories Inc.), transferred to ddPCR 96-well plates (cat. no. 12001925, Bio-Rad Laboratories Inc.) by pipetting and sealed with the PX1 PCR Plate Sealer (Bio-Rad Laboratories Inc.). The PCR reaction was performed using the DNA Engine Tetrad 2 Thermal Cycler (Bio-Rad Laboratories Inc.). The cycling steps were 95 °C 10 min; 40 cycles of (94 °C 30 s, 59.5 °C 1 min); 98 °C 10 min; 4 °C hold, with a ramp rate of 2 °C/s. The data were then visually inspected on the QuantaSoft Analysis v. 1.7.4.0917 and QuantaSoft Analysis Pro v. 1.0.596.0525 (Bio-Rad Laboratories Inc.) programs.

Each plate contained at least one no-template control for each assay to assess for putative contamination. All samples were run in duplicate.

#### 2.3.4. Data Extraction and Filtering

Droplet data were extracted as comma-separated values (CSV) files from the QuantaSoft Analysis software and imported into Microsoft Excel.

Subsequent filtering was performed in Microsoft Excel using a minimum threshold of 25 droplets for individual droplet categories and 8500 accepted droplets per reaction. Data from two successful wells were used in subsequent analyses. In cases of more than two successful wells for the same assay and sample, the two runs with the largest number of accepted droplets were used in the analysis. The data were grouped as normal, gain, and loss samples by the predicted *TTN* SD copy number category based on the respective NMD-CGH-array results.

The range, mean, standard deviation (σ), and coefficient of variation in percent (%CV) were extracted and calculated of the accepted droplet number and target copies per 20 μL, and for the estimated CN by ddPCR and aCGH for the respective regions targeted by the ddPCR assays.

### 2.4. Statistical Analysis

To assess the performance of the ddPCR assays in CN classification, we performed one-way analysis of variance (ANOVA) tests and post hoc Tukey’s honest significant difference (Tukey’s HSD) test using the ddPCR derived CN and the aCGH-predicted CNV class (either normal, loss, or gain). Additionally, we used Bland–Altman analysis [35] to assess the agreement between the method using the ddPCR derived CN and the aCGH-estimated CN.

A linear regression analysis was performed using the CN estimates from ddPCR and aCGH. In addition, the Pearson correlation coefficient was also calculated from these data. A Pearson correlation coefficient of >0.70 was considered a strong correlation.

To assess reproducibility within experiments, we performed intra-assay analyses separately for all assays using duplicates run within the same experiment. The analysis included calculations of %CV and σ of the differences between repeated measurements [35].

To assess repeatability between experiments, we performed inter-assay analyses separately for all assays using duplicates run on separate plates. The analysis included calculations of %CV and σ of the differences between repeated measurements [35].

The accuracy, sensitivity, and specificity of the assays were assessed as described in [36], by grouping the samples by their aCGH estimated CN for respective targeted region. For the *TTN* SD exon I assay, ddPCR CN values within the interval [7.5, 8.5] were considered normal. For the *TTN* SD exon VII and *NEB* TRI exon VIII assays, ddPCR CN values within the interval [5.5, 6.5] were considered normal. For the *TTN* Post-SD assay, ddPCR CN values between [1.5, 2.5] were considered normal. Values subceeding and exceeding the given intervals were classified as losses and gains as per the ddPCR results, respectively.

All statistical analyses were performed in RStudio (v.1.4.1103) using R v.4.0.4. [37,38]. Boxplots were generated using ggplot2 v.3.3.6 [39].

## 3. Results

### 3.1. Data Overview

Altogether 55 samples passed the initial quality filtering in all assays in at least two parallel wells. Of these, 36 were samples from neuromuscular disorder patients and 19 were samples from unaffected family members. Of all samples, 35 were classed as normal (CN = 6), 11 as losses (CN < 6), and 9 as gains (CN > 6) as per the CNV prediction of the NMD-CGH-array for the *TTN* SD region. Gains and losses in the *TTN* SD region were present in both patient and unaffected family member samples.

Table 1 shows the number of samples representing different predicted CN classes of the *TTN* SD and the CN distribution between affected and unaffected individuals. The expected CN of the *TTN* SD region as per the NMD-CGH-array data varied between 5 and 11 in the samples analysed. The expected CN of the *NEB* TRI region varied between 4 and 11, and an average CN of 6 was classified as normal for the *NEB* TRI also.

**Figure 2:**
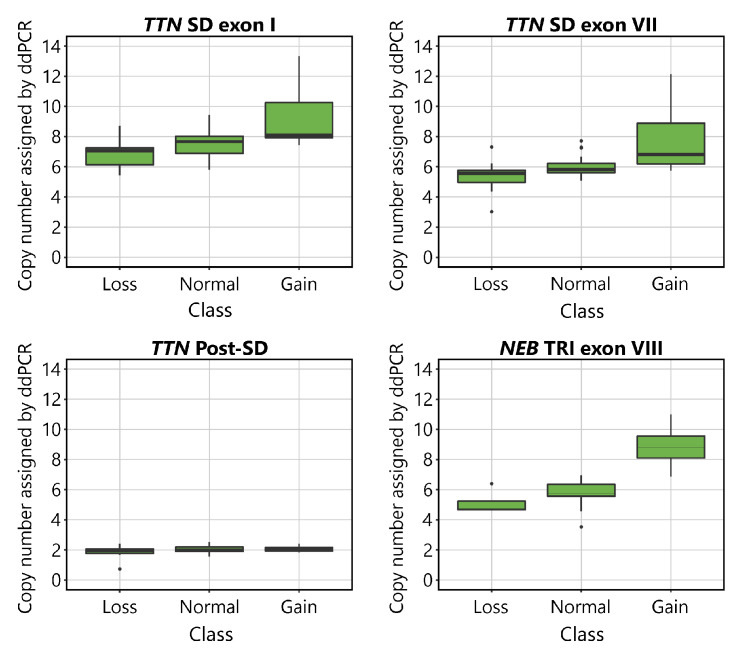
Boxplots visualising the CN of the *TTN* SD exon I, *TTN* SD exon VII, *TTN* Post-SD and *NEB* TRI exon VIII assays in relation to the CN assigned by aCGH to the *TTN* SD in the *TTN* targeting assays, and the *NEB* TRI in the *NEB* targeting assay. The normal CNs for the assays are 8 for *TTN* SD exon I, 6 for *TTN* SD exon VII, 2 for *TTN* Post-SD, and 6 for *NEB* TRI exon VIII. In the *TTN* targeting assays, 9 samples were categorised in the gain group, 35 in the normal group, and 11 in the loss group, according to their *TTN* SD CN estimated by the NMD-CGH-array. In the *NEB* TRI exon VIII assays, 4 samples were categorised in the gain group, 47 in the normal group, and 4 in the loss group, according to their *NEB* TRI CN as estimated by the NMD-CGH-array. The error bars represent the 95% confidence interval.

The CNs indicated by ddPCR are shown plotted against the aCGH-determined CN class for the *TTN* SD and *NEB* TRI in Figure 2. Visual inspection of the plots suggests that the assays detect differences between samples belonging to different CN groups. The mean values of the normal groups approach the expected values of 8 for the *TTN* SD exon I, 6 for *TTN* SD exon VII and *NEB* TRI exon VIII, and 2 for *TTN* Post-SD. As expected, differences were seen between groups in all assays except for the *TTN* Post-SD assay.

The mean accepted droplet count was 15,644 (σ = 2159.9, %CV = 13.8) for the *TTN* SD exon I assay, 15,501 (σ = 2322.3, %CV = 15.0) the *TTN* SD exon VII assay, 15,580 (σ = 2482.4, %CV = 15.9) for the *TTN* Post-SD assay, and 15,042 (σ = 2122.0, %CV = 14.1) for the *NEB* TRI exon VIII assay. The range, mean, σ, and %CV values for accepted droplet numbers, target, and reference copies per 20 μL are presented in Supplemental Table S3 broken down by the assigned CN groups. Droplet numbers showed no significant differences between assays or groups. The means of the target copies per 20 μL were distributed as expected, with the highest number of target copies in the *TTN* SD exon I assay, equal and lower number of target copies in the *TTN* SD exon VII and *NEB* TRI exon VIII assays, and the lowest number of target copies in the *TTN* Post-SD assay. The range, mean, σ, and %CV values for the ddPCR and aCGH estimated CNs for each ddPCR targeted sequence are presented in Supplemental Table S4.

The difference between the CNs estimated by the *TTN* SD exon I and *TTN* SD exon VII assays was normally distributed around the mean value of 1.61, [−0.85, 3.51]. The mean difference was marginally larger in the normal group (1.63, [−0.85, 3.51]) as compared with the gain (1.59, [1.11, 2.43]) and loss (1.55, [0.92, 2.50]) groups. In the normal group, the expected difference was 2.00, to account for the number of times the amplicon sequences are expected to occur in the genome.

### 3.2. One-Way ANOVA and Tukey’s HSD

The one-way ANOVA indicated statistically significant differences between the ddPCR results for at least two different groups in assays *TTN* SD exon I (F(2,52) = [9.80], *p* < 0.001), *TTN* SD exon VII (F(2,52) = [11.89], *p* < 0.00001), and *NEB* TRI exon VIII (F(2,52) = [32.95], *p* < 0.00001). No statistically significant differences were found between the groups in the *TTN* Post-SD assay (F(2,52) = [1.83], *p* = 0.171), as expected.

Tukey’s HSD test for multiple comparisons found that the mean value of the ddPCR results was significantly different between the normal and gain, and loss and gain groups in all assays except for the *TTN* Post-SD assay. No statistical significance was found between the normal and loss groups in any assays.

The complete ANOVA and Tukey’s HSD test results are presented in Supplemental Table S5.

### 3.3. Pearson Correlation

All Pearson correlation coefficients, except that for the *TTN* Post-SD assay against its corresponding *TTN* SD aCGH CN, exceeded the set threshold of 0.70 for a strong correlation. The Pearson correlation coefficients are presented in Table 2.

### 3.4. Linear Regression

The overall regression was statistically significant for the *TTN* SD exon I assay (adjusted R^2^ = 0.53, F(1,53) = 50.59, *p* < 0.00001), the *TTN* exon VII assay (adjusted R^2^ = 0.66, F(1,53) = 104.9, *p* < 0.00001), and the *NEB* TRI exon VIII assay (adjusted R^2^ = 0.67, F(1,53) = 113, *p* < 0.00001). The linear regression analysis found that the ddPCR assays seemed to recognise CNVs of the *TTN* SD region adequately. The *TTN* SD exon VII assay approached the already validated *NEB* TRI exon VIII assay [27] in its accuracy.

The overall regression of the *TTN* Post-SD assay CN estimates against the aCGH estimated CN of the *TTN* backbone (excluding the SD) was statistically significant (adjusted R^2^ = 0.07, F(1,53) = 5.1, *p* < 0.05). The adjusted R^2^ value approaching 0 indicates that the sequence targeted by the *TTN* Post-SD assay indeed lies outside the *TTN* SD region and that it adequately amplifies a diploid region. The overall regression of the *TTN* Post-SD assay CN estimates against the aCGH estimated CN of the *TTN* SD region was not statistically significant (adjusted R^2^ = −0.002, F(1,53) = 0.89, *p* = 0.351), which further supports this conclusion.

The overall regression of the *TTN* SD exon I assay CN estimates against the *TTN* SD exon VII assay CN estimates was statistically significant (adjusted R^2^ = 0.75, F(1,53) = 160.5, *p* < 0.00001), indicating concordance between the assays.

**Figure 3:**
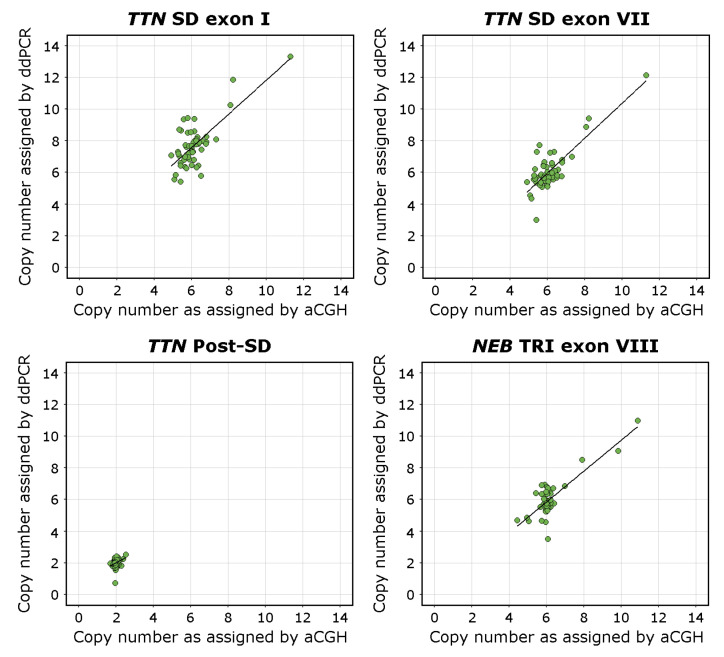
Scatterplots visualising the CN of the *TTN* SD exons I and VII, the *TTN* Post-SD target sequence, and *NEB* TRI exon VIII in relation to the CNs estimated using the NMD-CGH-array with corresponding linear regression trend lines. The aCGH CN estimate for the *TTN* Post-SD assay is derived from the calculated CN of the *TTN* backbone. The deviation from the 1-to-1 ratio between the NMD-CGH-array derived and *TTN* SD exon I assay derived CN estimates is consistent with the fact that the CN of exons I and II of the *TTN* SD is higher than than the average CN of the entire repeated block, which the NMD-CGH array cannot detect due to methodological limitations.

The complete results of the linear regression models are presented in Supplemental Table S6. Visualisations of the linear regression analyses and scatterplots of the data are presented in Figure 3.

### 3.5. Bland–Altman Analysis

**Figure 4:**
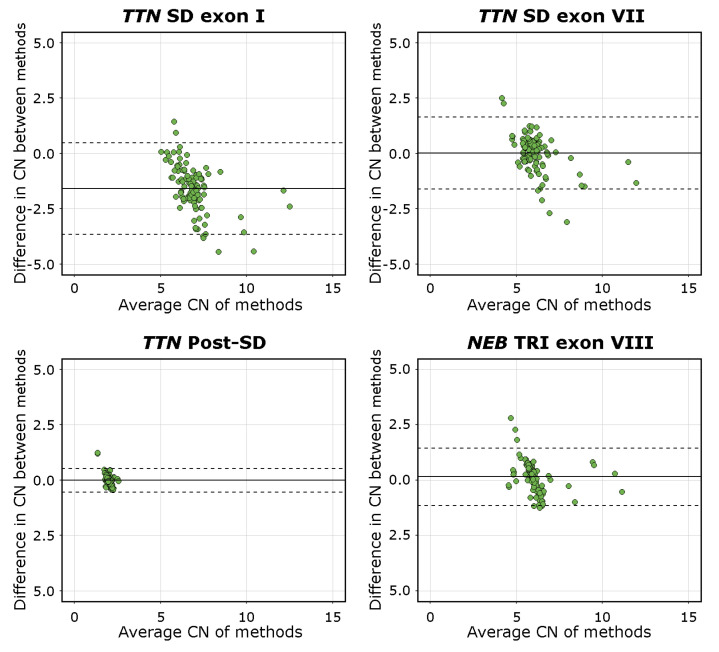
Bland-Altman plots of all assays. The solid line represents the mean difference, and the dashed lines represents the upper and lower limits of the 95% C.I. Mean values approaching 0 indicate a higher level of agreement between the compared methods, as seen for the *TTN* SD exon VII, *TTN* Post-SD, and *NEB* TRI exon VIII assays. The mean of differences of the *TTN* SD exon I assay deviates from 0 and approaches an absolute value of 2.0, which is the expected difference in CN estimates derived using the different methods.

The mean difference was −1.59 [95%C.I. −3.65, 0.48] for *TTN* SD exon I, 0.02 [95%C.I. −1.61, 1.65] for *TTN* SD exon VII, −0.01 [95%C.I. −0.54, 0.52] for *TTN* Post-SD, and 0.13 [95%C.I. −1.15, 1.45] for *NEB* TRI exon VIII. The visualisations of the Bland–Altman analysis are shown in Figure 4. Mean values approaching 0 indicate higher levels of agreement between the methods compared. The *TTN* SD exon VII, *TTN* Post-SD, and *NEB* TRI exon VIII assays, therefore, performed adequately, while the mean difference and scatter in differences in the *TTN* SD exon I results indicate a lower degree of agreement between the methods. This is consistent with the fact that the *TTN* SD exon I and exon II CN is higher than the average CN of the entire repeated block, which the NMD-CGH array cannot detect due to methodological limitations. The complete Bland–Altman analysis data, including 95%C.I. for the limits of agreement, are presented in Supplemental Table S7.

### 3.6. Intra-Assay and Inter-Assay Analyses

To assess the reproducibility of the assays, the intra-assay duplicate means, σ, and differences, and %CV were calculated along with mean %CV, mean difference ($\bar{\delta }$) and mean σ of differences ($\bar{\sigma }$) for all samples per assay. To assess the repeatability of the assays, the inter-assay duplicate means, σ, differences, and %CV were calculated along with mean %CV, $\bar{\delta }$, and $\bar{\sigma }$ of differences for all samples per assay.

The summarised intra-assay and inter-assay analysis results are presented in Table 3. The complete intra-assay and inter-assay analyses with underlying data are presented in Supplemental Tables S8 and S9, respectively.

### 3.7. Accuracy, Sensitivity, and Specificity

The *TTN* SD exon I assay yielded an accuracy of 0.218, a sensitivity of 0.667, and a specificity of 0.389. The *TTN* SD exon VII assay yielded an accuracy of 0.582, a sensitivity of 0.526, and a specificity of 0.639. The *TTN* Post-SD assay yielded an accuracy of 0.964 and a specificity of 0.964. Sensitivity for the *TTN* Post-SD assay could not be calculated due to the lack of both true positives and false negatives when assessed against the *TTN* backbone. The *NEB* TRI exon VIII assay yielded an accuracy of 0.691, a sensitivity of 0.875, and a specificity of 0.660.

## 4. Discussion

Our novel ddPCR assays confirm the presence of recurring CNVs within the *TTN* SD region, as first seen on the NMD-CGH-array [18]. CNVs in the *TTN* SD region are about three times more common than CNVs in the *NEB* TRI region. To our knowledge, this is the first time that CNVs within this region are acknowledged to this degree.

Our custom ddPCR assays for *TTN* SD exon I and *TTN* SD exon VII reliably recognise samples with gains in the *TTN* SD region, as supported by the statistical tests performed.

These gains were also detected by the NMD-CGH-array [18]. However, custom CGH-array methodology is challenged by the sequence length of the *TTN* SD region. Furthermore, the *TTN* SD is doubly repetitive; on top of the block repeating, the exons within the block are similar and share sequences with flanking exons. Therefore, the resolution and mathematical mode of analysis of aCGH data are challenged by this region, as it heavily relies on the possibility of incorporating enough unique and tiled probes, and aberration calls are typically made based on several consecutive affected probes.

There was no statistical significance between the normal and loss groups in the *TTN* SD exon I and *TTN* SD exon VII assays. This is presumably due to two factors: the relatively low number of samples and thus the lack of power in the statistical methods and the lack of CN amplitude variance within the deletion group. We have yet to see losses of two or more copies of the *NEB* TRI region in the over 430 samples that we have previously analysed on our custom NM- and NMD-CGH-arrays [17,18]. We have, however, seen cases with up to eight extra copies, yielding a total CN of 14 of the *NEB* TRI. It is also possible that at least part of the suspected *TTN* SD region losses are methodological artefacts due to the relatively low number of good quality aCGH probes that can be designed in the region. This, in turn, is limited by the repetitiveness and length of the *TTN* SD region.

The ddPCR method for detecting CNVs within the *TTN* SD could theoretically be improved by designing more assays covering other sequences of the region. In our experience, however, in silico optimisation does not necessarily guarantee working assays. Furthermore, the exons within the repeating block of the *TTN* SD are highly similar, limiting the possibilities for unique assay design.

Our study was also affected by the samples’ heterogeneous background in terms of DNA extraction method and storage; the method would most probably significantly benefit from streamlining the DNA extraction process. Different DNA extraction methods, the quality of the DNA, and possible amplification or fluorescent chemistry inhibitors may well affect the ddPCR assay results. It is difficult to conclude which factors are marginally influential and which affect the end result significantly with a variable sample collection. Our previous study observed that diversity in DNA extraction methods and quality of DNA does introduce a level of variability in the results [27].

The intra- and inter-assay analyses showed adequate reproducibility and acceptable repeatability. The larger repeatability %CV values may be explained by differences in manual handling, such as pipetting techniques between the persons who produced the data. Both intra- and inter-assay analysis results could improve by using automated pipetting machinery.

The *TTN* SD is part of the PEVK-encoding region of titin. This region undergoes extensive tissue-specific alternative splicing giving rise to cardiac and skeletal muscle-specific isoforms. The *TTN* SD exons seem to be missing from the cardiac isoforms [40]. The PEVK region forms an elastic spring that modulates titin-based force in skeletal muscle through interactions with calcium and actin [41].

What remains to be elucidated is the exact CN of the *TTN* SD gains and losses, along with the detailed structure and orientation of gains. Furthermore, phenotype-genotype correlation analyses are needed to pinpoint a pathogenic threshold of the *TTN* CNVs. It is unlikely that a single repeat block gain or loss would significantly alter the phenotype, seeing the size of the gene and its product. However, we hypothesise that a large enough size deviation of the PEVK region from normal may affect the elastic properties of titin and force generation in skeletal muscle.

Approximately 5% of the human genome consists of SD regions, and they are a significant feature of mammalian genomes [1,2,3,4]. Repetitive regions, including SDs, are often neglected in both MPS-based and aCGH methods. While sequencing technology and analysis of sequence data have come a long way in terms of CNV detection [14,15], the methodology has significant limitations regarding repetitive sequences. DNA amplification is a common step in MPS protocols, and in the case of repeated regions, it may severely distort the actual repeat number in the subsequent analysis. More importantly, the length of the contigs that modern MPS technologies produce is insufficient to get a reliable alignment of these repetitive regions, which may span several megabases.

Attempts to sequence over the *NEB* TRI and *TTN* SD regions have been unsuccessful, as even the long-read sequencing methods often rely on an amplification step. As long-read sequencing methods develop in due time, we hope to sequence over both the *NEB* TRI and *TTN* SD regions.

## Figures and Tables

**Table 1:** Number of samples from affected and unaffected individuals representing different predicted classes of the *TTN* SD according to the NMD-CGH-array. The normal CN for the *NEB* TRI region is 6; any deviation from this is considered either a loss or a gain.

					*NEB* TRI Status		
Group	*TTN* SD CN	Affected	Unaffected	Total	Loss	Normal	Gain
Loss	5	7	4	11	1	9	1
Normal	6	21	14	35	1	33	1
	7	6	0	6	2	3	1
Gain	8	2	0	2	0	1	1
	11	0	1	1	0	1	0
	Totals	36	19	55	4	47	4

**Table 2:** Pearson correlation coefficients for interpreted CN by ddPCR against NMD-CGH array derived CN estimates. A Pearson correlation coefficient of 0.70 was considered the lower threshold for a strong correlation.

Data 1	Data 2	Coefficient
*TTN* SD exon I ddPCR	*TTN* SD aCGH	0.731
*TTN* SD exon VII ddPCR	*TTN* SD aCGH	0.815
*TTN* Post-SD	*TTN* SD aCGH	0.128
*NEB* TRI exon VIII	*NEB* TRI	0.825
*TTN* SD exon I ddPCR	*TTN* SD exon VII ddPCR	0.867

**Table 3:** Intra- and inter-assay analysis results, including *n*, %CV, mean difference ($\bar{\delta }$) and mean σ ($\bar{\sigma }$) for each ddPCR assay.

	Intra-Assay Analysis				Inter-Assay Analysis			
Assay Target	*n*	%CV	$\bar{\delta }$	$\bar{\sigma }$	*n*	%CV	$\bar{\delta }$	$\bar{\sigma }$
*TTN* SD exon I	45	4.40	0.04	0.75	10	12.75	0.14	0.48
*TTN* SD exon VII	42	3.97	0.16	0.12	13	3.95	0.02	0.13
*TTN* Post-SD	40	2.31	0.00	0.01	15	4.29	0.06	0.05
*NEB* TRI exon VIII	42	3.77	0.05	0.06	12	3.51	0.05	0.16

## Data Availability

All relevant data are available in the Supplemental Material. Raw data are available from the authors at reasonable request.

## Supplementary Materials

The following are available online at https://www.mdpi.com/article/10.3390/genes13050905/s1, Table S1: dMIQE Checklist, Table S2: Primer and probe sequences and amplicon lengths and details, Table S3: Accepted droplet number and target copies per reaction, Table S4: Minimum, maximun, mean, σ and %CV of estimated copy number by ddPCR and aCGH, Table S5: Complete one-way ANOVA and Tukey’s HSD results, Table S6: Complete linear regression analysis results, Table S7: Complete Bland–Altman analysis results, Table S8: Intra-assay analysis results, Table S9: Inter-assay analysis results, Table S10: Complete filtered data.

## Abbreviations

The following abbreviations are used in this manuscript:

aCGH	array-Comparative Genomic Hybridisation
*AGO1*	Argonaute 1
ANOVA	Analysis of variance
CN	Copy number
CNV	Copy number variation
ddPCR	Droplet Digital PCR
*EIF2C1*	See *AGO1*
FAM	Fluorescein amitide
HEX	Hexachloro-fluorescein
HSD	Honest significant difference test
MPS	Massively parallel sequencing
*NEB*	Nebulin gene
PEVK	PEVK-rich domain of titin
SD	Segmental duplication
TRI	Triplicate region of nebulin
*TTN*	Titin gene
σ	Standard deviation
%CV	Coefficient of variation in %
