# Supplementary material for: Array Comparative Genomic Hybridisation and Droplet Digital PCR Uncover Recurrent Copy Number Variation of the TTN Segmental Duplication Region
Source: Genes (Basel). 2022 May 19;13(5):905. doi: 10.3390/genes13050905 (PMC9142044; doi:10.3390/genes13050905)
Supplement: Supplementary file 1 [file genes-13-00905-s001.zip › Tables S2-S9.pdf]

Supplemental Table S2

TTN SD exon I							
**Target**	**Sequence**	**Amplicon length**	**Amplicon locations within NG_011618.3**	**GC%**	**Length (bp)**	**Tm (°C)**	**Expected normal CN**
Forward primer	GTGCCACCCACAAAAGGTAG	115-116 bp	c.72355_172468, c.176616_176729, c.180876_180989, c.183524_183638	50	20	54,32	8
Reverse primer	TTGGCACCTCTGGGACTTTA			50	20	54,75	
Hydrolysis probe	TGCTGTGTTTGATATTGTTTGTTTGTC			33	27	56,87	
TTN SD exon VII							
**Target**	**Sequence**	**Amplicon length**	**Amplicon locations within NG_011618.3**	**GC%**	**Length (bp)**	**Tm (°C)**	**Expected normal CN**
Forward primer	AGCTCAAGAAGTTGTCCCAG	105 bp	c.173588_173692, c.177849_177953, c.182109_182213	50	20	55,2	6
Reverse primer	TCTGCAGAAAAAGGACAGGG			50	20	55,11	
Hydrolysis probe	AACCAGAAGCCCCACCTGCCACAGG			64	25	65,15	
TTN POST-SD							
**Target**	**Sequence**	**Amplicon length**	**Amplicon locations within NG_011618.3**	**GC%**	**Length (bp)**	**Tm (°C)**	**Expected normal CN**
Forward primer	GGGTGCTCTGAGCTTTTGTC	120 bp	c.183985_184104	55	20	56,8	2
Reverse primer	CGACAGCAGGTGCTTTCTTT			50	20	57,07	
Hydrolysis probe	ACCGTACCACCTAAGAAACCTGTCC			52	25	59,22	
NEB TRI exon VIII							
**Target**	**Sequence**	**Amplicon length**	**Amplicon locations within NG_009382.2**	**GC%**	**Length (bp)**	**Tm (°C)**	**Expected normal CN**
Forward primer	ACCACAAAGCCAAGATCTCC	104 bp	c.138874_138977, c.149427_149530, c.159978_160081	50	20	54,15	6
Reverse primer	GTGCAGGTAATGGCGATAGT			50	20	54,9	
Hydrolysis probe	CATGGTGTCCATCAGCGCTGCCAAAGAAGG			57	30	68,78	

Supplemental Table S3

	Droplet number per 20 µl					Target copies per 20 µl					Reference copies per 20 µl				
	Min	Max	Mean	σ	%CV	Min	Max	Mean	σ	%CV	Min	Max	Mean	σ	%CV
***TTN* SD exon I**	9823	20392	15651,3	2159,5	13,8	2478,6	101233,1	18267,2	20749,8	113,6	540,8	26111,1	4804,4	5306,4	110,4
**Loss group**	10024	20392	15423,2	2630,1	17,1	2756,5	85709,3	20664,4	21530,2	104,2	1031,5	23068,4	5797,1	5919,5	102,1
**Normal group**	10963	19903	15781,7	1972,0	12,5	2478,6	101233,1	20088,1	22396,8	111,5	756,2	26111,1	5240,5	5540,7	105,7
**Gain group**	9823	18823	15423,1	2319,3	15,0	3141,3	17818,4	8256,0	4980,9	60,3	540,8	4634,2	1895,3	1279,2	67,5
***TTN* SD exon VII**	8786	19674	15500,8	2322,3	15,0	1771,6	76381,2	15025,9	16904,7	112,5	687,1	26081,8	4940,6	5370,8	108,7
**Loss group**	10592	19146	14765,2	2490,1	16,9	1771,6	12761,5	16755,0	18378,6	109,7	994,9	21523,2	5794,9	5859,3	101,1
**Normal group**	8786	19674	15620,8	2179,2	14,0	2063,9	76381,2	16579,1	18021,7	108,7	706,0	26081,8	5434,4	5654,0	104,0
**Gain group**	9909	19207	15933,2	2585,9	16,2	2842,6	13495,9	6872,4	3551,3	51,7	687,1	4554,4	1976,1	1239,5	62,7
***TTN* Post-SD**	8774	19697	15464,3	2336,5	15,1	835,5	64894,7	6496,5	9596,2	147,7	971,2	66745,8	6423,1	9632,8	150,0
**Loss group**	10514	18473	14548,1	2508,0	17,2	938,9	64894,7	10781,5	17261,7	160,1	1059,6	66745,8	10960,7	17565,3	160,3
**Normal group**	8774	19697	15674,1	2382,1	15,2	835,5	33377,9	6076,6	6657,6	109,6	971,2	34255,9	5901,3	6435,3	109,0
**Gain group**	13722	18709	15768,1	1684,1	10,7	1139,5	9574,6	2892,3	2055,2	71,1	1059,4	9889,7	2906,7	2207,0	75,9
***NEB* TRI exon VIII**	9711	19358	15042,2	2122,0	14,1	1789,7	74472,9	15426,2	15795,6	102,4	670,8	28346,9	5223,1	5641,4	108,0
***TTN* Loss group**	11303	18539	15610,9	2219,9	14,2	2046,1	58604,0	19703,2	16115,9	81,8	1082,1	28346,9	7072,8	6610,0	93,5
***TTN* Normal group**	10024	19358	15113,1	2004,7	13,3	1839,4	74472,9	16535,2	16746,2	101,3	702,3	26359,4	5522,5	5685,6	103,0
***TTN* Gain group**	9711	17672	14351,0	2320,4	16,2	1789,7	13003,4	564,2	3944,9	699,3	670,8	4463,4	1798,2	1291,0	71,8
***NEB* Loss group**	12384	16497	15306,1	1429,3	9,3	2334,3	16497,0	3771,1	1728,8	45,8	974,7	2795,0	1501,3	750,5	50,0
***NEB* Normal group**	9711	19358	15013,7	2152,8	14,3	1789,7	74472,9	15595,2	15661,2	100,4	670,8	28346,9	5443,9	5790,7	106,4
***NEB* Gain group**	11852	18539	15113,1	2531,1	16,7	6957,7	53751,6	15113,1	2531,1	16,7	1504,1	15463,2	6351,4	5619,5	88,5

Supplemental Table S4

	ddPCR estimated CN					aCGH estimated CN				
***TTN* SD exon I**										
	Min	Max	Mean	σ	%CV	Min	Max	Mean	σ	%CV
Loss (n = 11)	4,99	8,85	7,08	1,07	15,16	4,90	5,79	5,37	0,24	4,42
Normal (n = 35)	5,42	10,59	7,63	1,00	13,15	5,34	7,31	6,06	0,34	5,55
Gain (n = 9)	5,05	13,68	8,84	2,29	25,91	6,50	11,29	7,50	1,56	20,86
***TTN* SD exon VII**										
	**ddPCR estimated CN**					**aCGH estimated CN**				
	Min	Max	Mean	σ	%CV	Min	Max	Mean	σ	%CV
Loss (n = 11)	2,90	7,54	5,45	1,03	18,87	4,90	5,79	5,37	0,24	4,42
Normal (n = 35)	4,96	9,46	5,99	0,77	12,81	5,34	7,31	6,06	0,34	5,55
Gain (n = 9)	5,28	12,62	7,36	2,10	28,53	6,50	11,29	7,50	1,56	20,86
***TTN* Post-SD**										
	**ddPCR estimated CN**					**aCGH estimated CN**				
	Min	Max	Mean	σ	%CV	Min	Max	Mean	σ	%CV
Loss (n = 11)	0,74	16,77	2,71	3,18	117,53	1,80	2,29	1,99	0,12	5,93
Normal (n = 35)	1,49	40,19	3,14	5,90	187,87	1,67	2,52	1,99	0,15	7,54
Gain (n = 9)	2,43	1,87	2,16	0,18	8,17	1,92	2,05	1,98	0,05	2,57
***NEB* TRI exon VIII**										
	**ddPCR estimated CN**					**aCGH estimated CN**				
	Min	Max	Mean	σ	%CV	Min	Max	Mean	σ	%CV
Loss (n = 4)	4,58	4,72	4,66	0,06	1,25	4,43	5,04	4,73	0,43	9,13
Normal (n = 47)	3,27	7,06	5,82	0,67	11,57	4,95	6,40	5,98	0,22	3,74
Gain (n = 4)	6,76	11,39	8,86	1,60	18,10	6,97	10,87	8,89	1,78	20,00

Supplemental Table S5

One-way ANOVA		
Assay	F	p
TTN SD exon I	F(2,52) = [9.797]	0.000245 ***
TTN SD exon VII	F(2,52) = [11.89]	5.58*10-5 ****
TTN Post-SD	F(2,52) = [1.826]	0.171
NEB TRI exon VIII	F(2,52) = [32.95]	5.72*10-10 ****

Tukey's HSD									
Assay	Loss-Gain			Norm-Gain			Norm-Loss		
	Mean difference	95% C.I.	p	Mean difference	95% C.I.	p	Mean difference	95% C.I.	p
TTN SD exon I	-2.3142	[-3.62, -1.01]	0.00023 ***	-1.6458	[-2.73, -0.56]	0.0016549 **	0.6634	[-0.33, 1.67]	0.2507677
TTN SD exon VII	-2.2705	[-3.44, 1.09]	0.0000655 ***	-1.6854	[-2.66, -0.71]	0.0003466 ***	0.5851	[-0.32, 1.49]	0.2712789
TTN Post-SD	-0.1953	[-0.49, 0.09]	0.2444643	-0.0319	[-0.27, 0.21]	0.9452915	0.1634	[-0.06, 0.39]	0.1907538
NEB TRI exon VIII	-3.7188	[-5.00, -2.44]	0.0000000 ****	-3.0332	[-3.98, -2.09]	0.0000000 ****	0.6855	[-0.26, 1.63]	0.1950505

Supplemental Table S6

TTN SD exon I - aCGH data (*TTN* SD)					
Residuals	Min	1Q	Median	3Q	Max
	-1,37123	-0,30151	-0,02921	0,31195	2,3523
Coefficients	Estimate	Std.Error	t value	Pr(>|t|)	
(Intercept)	2,24924	0,50442	4,459	4,31E-06	
Actual	0,5021	0,06445	7,79	2,42E-10	
Residual standard error	0,6552	on 53 degrees of freedom			
Multiple R-squared	0,5338				
Adjusted R-squared	0,525				
F-statistic	50,59	on 1 and 53 degrees of freedom			
p-value	2,42E-10				

TTN SD exon VII - aCGH data (*TTN* SD)					
Residuals	Min	1Q	Median	3Q	Max
	-1,5155	-0,324	0,081	0,3046	1,5255
Coefficients	Estimate	Std.Error	t value	Pr(>|t|)	
(Intercept)	2,43729	0,36713	6,639	1,72E-08	
Actual	0,60341	0,05892	10,242	3,62E-14	
Residual standard error	0,5559	on 53 degrees of freedom			
Multiple R-squared	0,6643				
Adjusted R-squared	0,658				
F-statistic	104,9	on 1 and 53 degrees of freedom			
p-value	3,63E-14				

TTN Post-SD - aCGH data (*TTN* SD)					
Residuals	Min	1Q	Median	3Q	Max
	-1,2984	-0,433	-0,0933	0,1306	5,2061
Coefficients	Estimate	Std.Error	t value	Pr(>|t|)	
(Intercept)	5,2194	0,9636	5,417	0,00000151	
Actual	0,4488	0,477	0,941	0,351	
Residual standard error	0,9517	on 53 degrees of freedom			
Multiple R-squared	0,01643				
Adjusted R-squared	-0,002126				
F-statistic	0,8855	on 1 and 53 degrees of freedom			
p-value	0,351				

NEB TRI exon VIII - aCGH data (*NEB* TRI)					
Residuals	Min	1Q	Median	3Q	Max
	-1,05716	.0,33265	0,00188	0,22779	1,65442
Coefficients	Estimate	Std.Error	t value	Pr(>|t|)	
(Intercept)	1,95795	0,40098	4,883	1,00E-05	
Actual	0,69829	0,06579	10,614	1,01E-14	
Residual standard error	0,5358	on 53 degrees of freedom			
Multiple R-squared	0,6801				
Adjusted R-squared	0,674				
F-statistic	112,7	on 1 and 53 degrees of freedom			
p-value	1,01E-14				

TTN SD exon I - *TTN* SD exon VII					
Residuals	Min	1Q	Median	3Q	Max
	-2,37816	-0,29764	0,00002	0,37488	1,89014
Coefficients	Estimate	Std.Error	t value	Pr(>|t|)	
(Intercept)	2,00785	0,45944	4,37	5,82E-05	
Actual	0,93402	0,07373	12,67	2,00E-16	
Residual standard error	0,6957	on 53 degrees of freedom			
Multiple R-squared	0,7517				
Adjusted R-squared	0,747				
F-statistic	160,5	on 1 and 53 degrees of freedom			
p-value	2,20E-16				

Supplemental Table S6

TTN Post-SD - aCGH data (backbone)					
Residuals	Min	1Q	Median	3Q	Max
	-0,31169	-0,06439	-0,02439	0,03765	0,45462
Coefficients	Estimate	Std.Error	t value	Pr(>|t|)	
(Intercept)	1,70718	0,12623	13,524	<2e-16	
Actual	0,1414	0,06248	2,263	0,0278	
Residual standard error	0,1247	on 53 degrees of freedom			
Multiple R-squared	0,08811				
Adjusted R-squared	0,07091				
F-statistic	5,121	on 1 and 53 degrees of freedom			
p-value	0,02776				

Supplemental Table S7

	TTN SD exon I	TTN SD exon VII	TTN Post-SD	NEB TRI VIII
Number of comparisons	110	110	110	110
Maximum value for average measures	12,485	11,954	2,544	11,130
Minimum value for average measures	5,028	4,152	1,337	4,538
Maximum value for difference in measures	1,447	2,502	1,235	2,804
Minimum value for difference in measures	-4,446	-3,100	-0,446	-1,245
Bias	-1,587	0,018	-0,012	0,149
Standard deviation of bias	1,054	0,831	0,269	0,661
Standard error of bias	0,100	0,079	0,026	0,063
Standard error for limits of agreement	0,172	0,136	0,044	0,108
Bias- upper 95% CI	-1,388	0,175	0,039	0,274
Bias- lower 95% CI	-1,786	-0,139	-0,063	0,024
Upper limit of agreement	0,478	1,647	0,516	1,445
Upper LOA- upper 95% CI	0,819	1,916	0,603	1,659
Upper LOA- lower 95% CI	0,137	1,378	0,429	1,231
Lower limit of agreement	-3,652	-1,611	-0,540	-1,147
Lower LOA- upper 95% CI	-3,311	-1,342	-0,453	-0,932
Lower LOA- lower 95% CI	-3,994	-1,880	-0,627	-1,361
Mean of differences/means	-22,208	1,345	0,313	3,066
Point estimate of bias as proportion of lowest average	-31,568	0,436	-0,895	3,289
Point estimate of bias as proportion of highest average	-12,714	0,152	-0,471	1,341
Spread of data between lower and upper LoAs	4,130	3,258	1,056	2,592
Bias as proportion of LoA spread	-38,431	0,556	-1,134	5,758

Supplemental Table S8

TTN SD exon I					
Sample	CN Rep1	CN Rep2	Mean	σ	%CV
1423	7,59	7,64	7,62	0,04	0,46
1803	6,30	6,42	6,36	0,08	1,33
2421	5,43	6,24	5,84	0,57	9,82
2422	8,72	8,30	8,51	0,30	3,49
2423	6,85	7,34	7,10	0,35	4,88
2813	5,34	5,52	5,43	0,13	2,34
3223	7,81	6,76	7,29	0,74	10,19
3344	7,47	5,42	6,45	1,45	22,49
3531	9,09	7,39	8,24	1,20	14,59
3532	7,79	8,25	8,02	0,33	4,06
3533	7,77	7,67	7,72	0,07	0,92
3611	10,59	8,17	9,38	1,71	18,24
3612	5,79	7,43	6,61	1,16	17,54
3813	7,04	6,64	6,84	0,28	4,14
4523	8,59	8,60	8,60	0,01	0,08
4533	7,87	8,32	8,10	0,32	3,93
4543	7,87	7,75	7,81	0,08	1,09
4551	7,33	7,75	7,54	0,30	3,94
4552	7,62	7,71	7,67	0,06	0,83
4553	8,23	8,31	8,27	0,06	0,68
4583	12,62	11,08	11,85	1,09	9,19
4673	7,92	7,88	7,90	0,03	0,36
4722	7,41	7,21	7,31	0,14	1,93
4751	9,32	9,40	9,36	0,06	0,60
4752	6,18	6,68	6,43	0,35	5,50
4753	7,55	8,42	7,99	0,62	7,70
4762	7,84	7,97	7,91	0,09	1,16
4763	7,28	7,60	7,44	0,23	3,04
4793	7,04	7,08	7,06	0,03	0,40
4813	7,05	7,34	7,20	0,21	2,85
4821	6,24	6,29	6,27	0,04	0,56
4822	6,82	6,77	6,80	0,04	0,52
4823	7,62	7,75	7,69	0,09	1,20
4843	7,20	7,43	7,32	0,16	2,22
4844	7,90	8,09	8,00	0,13	1,68
4851	13,68	12,96	13,32	0,51	3,82
4873	7,69	7,91	7,80	0,16	1,99
4883	8,23	7,96	8,10	0,19	2,36
4891	7,94	9,16	8,55	0,86	10,09
4892	9,43	9,43	9,43	0,00	0,00
4893	7,11	6,90	7,01	0,15	2,12
4913	8,85	8,46	8,66	0,28	3,19
4933	8,75	8,69	8,72	0,04	0,49
5153	7,05	7,51	7,28	0,33	4,47
6003	6,22	6,72	6,47	0,35	5,46
**Mean %CV**					4,40
**Mean of differences**					0,04
**σ of differences**					0,75
**Total samples**					45

TTN SD exon VII					
Sample	CN Rep1	CN Rep2	Mean	σ	%CV
1803	5,71	5,93	5,82	0,15	2,59
2421	4,37	4,35	4,36	0,02	0,36
2422	6,57	6,35	6,46	0,15	2,38
2423	5,29	5,47	5,38	0,13	2,37
2813	2,90	3,14	3,02	0,17	5,52
3223	5,43	5,00	5,22	0,30	5,80
3344	9,46	5,13	7,30	3,07	42,01
3532	6,02	5,64	5,83	0,27	4,56
3533	5,80	5,93	5,86	0,09	1,52
3611	6,04	5,70	5,87	0,24	4,11
3612	5,63	5,75	5,69	0,09	1,50
3813	5,47	5,47	5,47	0,00	0,06
4523	7,29	7,20	7,25	0,06	0,86
4533	6,16	6,75	6,45	0,42	6,43
4543	5,81	5,86	5,83	0,03	0,56
4551	5,58	5,68	5,63	0,07	1,33
4552	5,79	5,82	5,80	0,03	0,47
4553	6,88	6,74	6,81	0,10	1,46
4583	9,14	9,69	9,42	0,39	4,14
4673	6,06	5,83	5,94	0,16	2,69
4722	5,62	5,49	5,56	0,10	1,72
4751	8,26	7,17	7,72	0,78	10,06
4752	5,38	5,36	5,37	0,02	0,36
4753	6,04	6,32	6,18	0,20	3,17
4763	5,84	5,82	5,83	0,02	0,28
4793	5,57	5,68	5,63	0,08	1,42
4813	6,01	5,65	5,83	0,25	4,36
4821	5,05	5,12	5,08	0,05	0,98
4822	5,26	5,14	5,20	0,08	1,62
4823	5,93	6,15	6,04	0,15	2,52
4843	5,71	5,65	5,68	0,04	0,71
4844	6,10	5,87	5,99	0,16	2,65
4851	12,62	11,67	12,14	0,67	5,54
4873	5,93	5,58	5,75	0,24	4,24
4883	7,25	6,72	6,99	0,38	5,42
4891	6,33	6,36	6,35	0,02	0,33
4892	5,61	7,22	6,42	1,14	17,83
4893	5,38	5,35	5,37	0,02	0,38
4913	7,54	7,09	7,31	0,32	4,36
4933	6,34	6,10	6,22	0,17	2,78
5153	5,46	5,38	5,42	0,05	0,93
6003	5,28	4,96	5,12	0,23	4,41
**Mean %CV**					3,97
**Mean of differences**					0,16
**σ of differences**					0,12
**Total samples**					42

Supplemental Table S8

TTN Post-SD					
Sample	CN Rep1	CN Rep2	Mean	σ	%CV
1803	2,34	2,37	2,36	0,02	0,90
2421	1,70	1,69	1,70	0,01	0,42
2422	2,23	2,25	2,24	0,01	0,63
2423	2,19	2,15	2,17	0,03	1,30
2813	0,75	0,72	0,74	0,02	2,89
3223	1,83	1,86	1,85	0,02	1,15
3344	2,21	2,13	2,17	0,06	2,61
3531	2,08	1,86	1,97	0,16	7,90
3532	1,90	1,88	1,89	0,01	0,75
3533	1,92	1,94	1,93	0,01	0,73
3612	1,95	1,94	1,95	0,01	0,36
3613	1,89	1,92	1,91	0,02	1,11
3813	1,86	2,13	2,00	0,19	9,57
4533	2,29	2,16	2,23	0,09	4,13
4543	1,95	1,95	1,95	0,00	0,00
4552	1,95	2,00	1,98	0,04	1,79
4553	1,80	1,86	1,83	0,04	2,32
4583	2,14	2,16	2,15	0,01	0,66
4584	2,10	1,92	2,01	0,13	6,33
4653	2,08	2,06	2,07	0,01	0,68
4673	2,08	2,01	2,05	0,05	2,42
4722	1,90	2,00	1,95	0,07	3,63
4751	2,23	2,43	2,33	0,14	6,07
4753	1,92	1,93	1,93	0,01	0,37
4762	2,34	2,37	2,36	0,02	0,90
4793	1,92	1,97	1,95	0,04	1,82
4813	1,94	1,99	1,97	0,04	1,80
4821	1,70	1,74	1,72	0,03	1,64
4822	1,82	1,77	1,80	0,04	1,97
4823	1,76	1,84	1,80	0,06	3,14
4843	1,99	1,99	1,99	0,00	0,00
4844	1,89	2,01	1,95	0,08	4,35
4851	1,91	1,94	1,93	0,02	1,10
4873	1,87	1,95	1,91	0,06	2,96
4883	2,15	2,16	2,16	0,01	0,33
4891	1,93	1,89	1,91	0,03	1,48
4893	2,01	2,06	2,04	0,04	1,74
4913	2,47	2,36	2,42	0,08	3,22
4933	2,23	2,14	2,19	0,06	2,91
5153	2,10	1,98	2,04	0,08	4,16
**Mean %CV**					2,31
**Mean of differences**					0,00
**σ of differences**					0,01
**Total samples**					40

NEB TRI exon VIII					
Sample	CN Rep1	CN Rep2	Mean	σ	%CV
1423	5,42	5,07	5,25	0,25	4,72
1803	6,82	7,06	6,94	0,17	2,45
2421	4,13	5,02	4,58	0,63	13,76
2422	6,45	6,27	6,36	0,13	2,00
2423	5,89	5,83	5,86	0,04	0,72
2813	3,27	3,78	3,53	0,36	10,23
3223	4,67	4,58	4,63	0,06	1,38
3344	6,19	6,71	6,45	0,37	5,70
3532	5,43	5,86	5,65	0,30	5,39
3533	5,98	5,56	5,77	0,30	5,15
3611	5,79	5,74	5,77	0,04	0,61
3612	4,74	4,58	4,66	0,11	2,43
3813	5,77	5,64	5,71	0,09	1,61
4523	6,62	6,97	6,80	0,25	3,64
4533	6,07	5,94	6,01	0,09	1,53
4543	5,44	5,77	5,61	0,23	4,16
4551	5,44	5,50	5,47	0,04	0,78
4552	5,66	6,07	5,87	0,29	4,94
4553	5,48	5,26	5,37	0,16	2,90
4583	8,89	8,15	8,52	0,52	6,14
4673	5,90	5,98	5,94	0,06	0,95
4722	5,40	5,66	5,53	0,18	3,32
4751	6,95	6,76	6,86	0,13	1,96
4752	9,00	9,15	9,08	0,11	1,17
4753	11,39	10,57	10,98	0,58	5,28
4762	6,26	6,48	6,37	0,16	2,44
4763	4,72	4,65	4,69	0,05	1,06
4793	5,00	4,70	4,85	0,21	4,37
4813	5,49	5,88	5,69	0,28	4,85
4821	5,22	5,31	5,27	0,06	1,21
4822	5,27	5,44	5,36	0,12	2,24
4823	5,35	5,26	5,31	0,06	1,20
4843	6,00	5,80	5,90	0,14	2,40
4844	5,77	5,87	5,82	0,07	1,21
4851	6,02	5,36	5,69	0,47	8,20
4873	5,83	6,12	5,98	0,21	3,43
4883	6,59	6,20	6,40	0,28	4,31
4893	6,53	6,19	6,36	0,24	3,78
4913	6,79	6,65	6,72	0,10	1,47
4933	6,16	5,92	6,04	0,17	2,81
5153	6,10	5,44	5,77	0,47	8,09
6003	7,06	5,93	6,50	0,80	12,30
**Mean %CV**					3,77
**Mean of differences**					0,05
**σ of differences**					0,06
**Total samples**					42

Supplemental Table S9

TTN SD exon I					
Sample	CN Rep1	CN Rep2	Mean	σ	%CV
1531	6,16	4,99	5,58	0,83	14,84
3613	8,28	7,75	8,02	0,37	4,68
4584	8,88	11,62	10,25	1,94	18,90
4653	7,54	6,04	6,79	1,06	15,62
4683	7,52	8,06	7,79	0,38	4,90
4693	6,69	5,96	6,33	0,52	8,16
4713	7,9	7,96	7,93	0,04	0,54
4721	8,05	6,33	7,19	1,22	16,92
4743	5,74	8,17	6,96	1,72	24,71
4761	6,55	5,05	5,80	1,06	18,29
**Mean %CV**					12,75
**Mean of differences**					0,14
**σ of differences**					0,48
**Total samples**					10

TTN SD exon VII					
Sample	CN Rep1	CN Rep2	Mean	σ	%CV
1423	5,37	5,47	5,42	0,07	1,26
1531	4,67	4,43	4,55	0,17	3,78
3531	5,30	5,85	5,57	0,39	6,94
3613	5,67	5,79	5,73	0,09	1,50
4584	9,51	8,27	8,89	0,87	9,81
4653	5,50	5,85	5,68	0,25	4,38
4683	6,82	6,41	6,62	0,29	4,36
4693	5,69	5,72	5,70	0,02	0,39
4713	6,79	6,47	6,63	0,23	3,40
4721	6,55	6,76	6,66	0,14	2,16
4743	5,62	5,76	5,69	0,09	1,66
4761	5,28	6,05	5,66	0,55	9,66
4762	6,01	6,19	6,10	0,12	2,03
**Mean %CV**					3,95
**Mean of differences**					-0,02
**σ of differences**					0,13
**Total samples**					13

TTN Post-SD					
Sample	CN Rep1	CN Rep2	Mean	σ	%CV
1423	1,78	1,78	1,78	0,00	0,00
1531	1,83	1,85	1,84	0,01	0,77
3611	1,95	1,72	1,84	0,16	8,86
4523	2,34	2,32	2,33	0,01	0,61
4551	1,49	1,63	1,56	0,10	6,35
4683	2,45	2,28	2,37	0,12	5,08
4693	2,47	2,57	2,52	0,07	2,81
4713	2,38	2,43	2,41	0,04	1,47
4721	2,01	1,87	1,94	0,10	5,10
4743	2,37	1,85	2,11	0,37	17,43
4752	1,60	1,77	1,69	0,12	7,13
4761	2,29	2,18	2,24	0,08	3,48
4763	2,30	2,19	2,25	0,08	3,46
4892	1,90	1,87	1,89	0,02	1,13
6003	2,00	1,98	1,99	0,01	0,71
**Mean %CV**					4,29
**Mean of differences**					0,06
**σ of differences**					0,05
**Total samples**					15

NEB TRI exon VIII					
Sample	CN Rep1	CN Rep2	Mean	σ	%CV
1531	5,58	5,48	5,53	0,07	1,28
3531	5,23	5,51	5,37	0,20	3,69
3613	5,31	5,92	5,62	0,43	7,68
4584	5,24	5,99	5,62	0,53	9,44
4653	5,77	5,79	5,78	0,01	0,24
4683	6,61	6,66	6,64	0,04	0,53
4693	6,87	6,98	6,93	0,08	1,12
4713	5,93	6,83	6,38	0,64	9,97
4721	5,90	5,69	5,80	0,15	2,56
4743	5,60	5,60	5,60	0,00	0,00
4761	6,84	6,59	6,72	0,18	2,63
4891	5,72	5,49	5,61	0,16	2,90
4892	6,96	5,73	6,35	0,87	13,71
**Mean %CV**					3,51
**Mean of differences**					-0,05
**σ of differences**					0,16
**Total samples**					12
